# Estimating the prevalence of alcohol-related disorders and treatment utilization in Bremen 2016/2017 through routine data linkage

**DOI:** 10.3389/fpsyt.2023.1002526

**Published:** 2023-01-26

**Authors:** Justin Möckl, Christina Lindemann, Jakob Manthey, Bernd Schulte, Jens Reimer, Oliver Pogarell, Ludwig Kraus

**Affiliations:** ^1^Department of Epidemiology and Diagnostics, Institut für Therapieforschung (IFT), Centre for Mental Health and Addiction Research, Munich, Germany; ^2^Department of Psychiatry and Psychotherapy, University Hospital, Ludwig-Maximilians-Universität Munich, Munich, Germany; ^3^Department of Psychiatry and Psychotherapy, Center for Interdisciplinary Addiction Research, University Medical Center Hamburg-Eppendorf, Hamburg, Germany; ^4^Department of Medical Psychology, Center for Health Care Research, University Medical Center Hamburg-Eppendorf, Hamburg, Germany; ^5^Department of Psychiatry, Medical Faculty, University of Leipzig, Leipzig, Germany; ^6^Zentrum für Psychosoziale Medizin, Klinikum Itzehoe, Itzehoe, Germany; ^7^Department of Public Health Sciences, Centre for Social Research on Alcohol and Drugs, Stockholm University, Stockholm, Sweden; ^8^Institute of Psychology, Eötvös Loránd University (ELTE), Budapest, Hungary

**Keywords:** alcohol dependence, treatment utilization, data linkage, routine data, epidemiology

## Abstract

**Background:**

In Germany, most individuals with alcohol dependence are recognized by the health care system and about 16% per year receive addiction-specific care. This paper aimed to analyze the prevalence and treatment utilization rate of people with alcohol dependence by type of addiction-specific care in the federal state of Bremen using routine and survey data.

**Methods:**

The number of individuals with alcohol dependence was estimated using data from the 2018 Epidemiological Survey of Substance Abuse (ESA). Furthermore, linked routine data of two statutory health insurances (SHIs), the German pension insurance (GPI), and the communal hospital group Gesundheit Nord – Bremen Hospital Group (GeNo), from 2016/2017, were analyzed. Based on SHI data, the administrative prevalence of various alcohol-related diagnoses according to the International Classification of Diseases (ICD-10), in various treatment settings, was extrapolated to the total population of Bremen. Based on all routine data sources, treatment and care services for individuals with alcohol dependence were also extrapolated to Bremen’s total population. Care services included outpatient addiction care visits and addiction-specific treatments, [i.e., qualified withdrawal treatment (QWT), outpatient pharmacotherapy as relapse prevention, and rehabilitation treatment].

**Results:**

Of the survey-estimated 15,792 individuals with alcohol dependence in Bremen, 72.6% (*n* = 11,467) had a diagnosis documented with an ICD-10 code for alcohol dependence (F10.2) or withdrawal symptoms (F10.3–F10.4). One in ten individuals with alcohol dependence (*n* = 1,689) used one or more addiction-specific care services during the observation period. Specifically, 4.3% (*n* = 675) received outpatient addiction care, 4.7% (*n* = 736) initiated QWT, 0.8% (*n* = 133) received pharmacotherapy, and 3.9% (*n* = 614) underwent rehabilitation treatment. The share of seeking addiction-specific treatment after diagnosis was highest among younger and male patients.

**Conclusion:**

Although more than half of the individuals with alcohol dependence are documented in the health system, utilization rates of addiction-specific treatments are low. These low utilization rates suggest that there are existing barriers to transferring patients with alcohol dependence into addiction-specific care. Strengthening primary medical care provision in dealing with alcohol-related disorders and improving networking within the addiction support system appear to be particularly appropriate.

## 1. Introduction

Germany has one of the highest alcohol consumption rates worldwide, with a per capita consumption of 10.6 L of pure alcohol for citizens aged 15 and older in 2019 (1, 2). Survey estimates based on the criteria of the Diagnostical and Statistical Manual (DSM-IV), reported a prevalence of 2.8% (1.4 million individuals aged 18–64 years) for alcohol abuse and 3.1% (1.6 million) for alcohol dependence (3). Furthermore, diagnoses for mental and behavioral disorders due to alcohol (F10.X) according to the International Classification of Diseases (ICD-10) were the third most frequent main diagnosis for inpatient hospitalization in men in 2018 (4).

Multiple treatment options for risky, harmful, and dependent alcohol use are discussed in the current German S3 guideline on “Screening, diagnosis, and treatment of alcohol-related disorders.” The goal of the guideline is to give recommendations for professionals as well as people affected by alcohol use and was developed by experts based on the available evidence (5). The recommendations include brief interventions as well as medical rehabilitation and other forms of post-acute treatment. Addiction rehabilitation or other post-acute treatment for alcohol dependence should be preceded by either physical detoxification or qualified withdrawal treatment (QWT). QWT is a German-specific term for an extended withdrawal treatment program, during which psychological and somatic concomitant and secondary diseases are considered, and further treatment for the underlying alcohol dependence is initiated using psychosocial interventions (5). Despite existing evidence-based procedures for early detection, adequate diagnosis, and the treatment of alcohol-related disorders (6) as well as the integration of different care systems (7, 8), the treatment rates for alcohol use disorders as compared to other mental illnesses are among the lowest globally (9, 10). The main reasons for this gap are, among others, structural barriers (8, 11), insufficient qualifications of doctors in alcohol dependence treatment (12), lack of patient motivation (13), and social stigma (14).

An international meta-analysis based on 12-month and lifetime treatment studies estimated the global treatment rate for alcohol use disorders at 17.3%, when informal support services, such as Alcoholics Anonymous, were also considered (9). A study including six European countries that only accounted for treatments offered by health professionals reported a similar treatment rate of 17.6% (15). In Germany in 2012, about one-third of individuals with alcohol dependence were identified by general practitioners, but in the same period, only about 16% were treated in hospital or outpatient addiction care, with 1.8% receiving rehabilitation treatment (16). A recent study also reported undertreatment of individuals with risky alcohol use and severe alcohol use disorder (17).

Previous estimates of treatment rates in Germany were based on diagnoses from aggregated data, such as hospital diagnosis statistics and the diagnosis portal of the Central Institute of Statutory Health Insurance Physicians [Zentralinstitut für die kassenärztliche Versorgung (Zi)] (16), were derived from per capita consumption (17) or based on survey data (15). In addition, several studies reported differences in diagnoses depending on the setting and the diagnostic instrument (18, 19). A study among primary care patients showed that general practitioners diagnose alcohol dependence more often in male and older patients compared to a standardized, self-administered closed-ended clinical diagnostic questionnaire (18).

The data linkage was conducted within the project “Implementation and evaluation of the guideline on screening, diagnosis and treatment of alcohol-related disorders” (IMPELA) in the federal state of Bremen (20). The aims of the present analysis were,

(1) To identify individuals with diagnoses of alcohol-related disorders in various treatment settings (administrative prevalence, i.e., prevalence of a specific disorder in a population based on routine data) as well as addiction-specific treatments and care of individuals with alcohol dependence in the routine data.
(2) Extrapolation of
  (a) The overall prevalence of individuals with alcohol dependence from survey data to the total population of Bremen.
  (b) The number of individuals with alcohol dependence and their addiction-specific treatments and care identified in the routine data to the total population of Bremen, and
(3) To estimate addiction-specific treatment rates for individuals with alcohol dependence in the total population of Bremen.

## 2. Materials and methods

The methods section is structured based on the different data sets and estimates. First, the study population of the linked routine data is introduced (section “2.1. Study population”), followed by the definition of identified diagnoses and treatments as well as the total populations for each routine dataset separately (sections “2.1.1. Statutory health insurance: Diagnoses,” “2.1.2. Statutory health insurance: Treatments,” and “2.1.3. Hospital group Gesundheit Nord: Outpatient addiction care”). In section “2.2. Survey data,” the use of the survey data and in section “2.3. Overall prevalence of alcohol dependence in Bremen” the estimation of the overall prevalence of alcohol dependence in Bremen is described. Finally, the extrapolation of the administrative prevalence together with the treatments in each data set (section “2.4. Administrative prevalence and extrapolation”) as well as the calculation of treatment rates in the total population of Bremen is explained (section “2.5. Treatment rate”). The notation used for the methods defines *N*/*n* as the empirical sample and population sizes, whereas *N̂*/*n̂* represent the estimated and extrapolated population sizes.

### 2.1. Study population

In Germany, health insurance is mandatory and consists of public statutory health insurances (SHIs) and private health insurance. Most people (90%) are insured by one of the SHIs, which cover medical treatments approved by the Joint Federal Committee (21). Depending on the type of treatment, different insurances are responsible for the reimbursement of the treatment costs. For example, withdrawal treatment is covered by health insurance funds, whereas rehabilitation treatment in most cases is financed by the German pension insurance (Deutsche Rentenversicherung; hereafter: GPI) (5). Eligibility criteria for the coverage of rehabilitation treatment by the GPI are, among others, having paid for the insurance for at least 6 months over the past 2 years and not being a civil servant. As the treatment goal is reintegration into the labor market, pensioners are not covered by the GPI. In this study three routine data sources were linked, these included data on diagnoses and treatments in public health care (inpatient and outpatient settings), addiction-specific care (outpatient addiction care), and rehabilitation.

To this end, regional master data and service data from 2016 and 2017 from (1) two SHIs in Bremen (AOK Bremen/Bremerhaven and hkk), (2) on outpatient addiction care services data of the communal hospital group Gesundheit Nord–Bremen Hospital Group (GeNo) in Bremen, and (3) the regional GPI (Deutsche Rentenversicherung Oldenburg-Bremen) were linked on an individual level (20). The individuals included in the study population were 16 years or older in 2016 and 2017, were living in Bremen or Bremerhaven; and had a main or secondary diagnosis of mental as well as behavioral disorders due to alcohol (F10.X) or had another fully alcohol-attributable diagnosis according to documentation of one of the three data sources mentioned above. A detailed list of all relevant diagnoses, as well as a description of the study population and the known total populations, are presented in Table 1 and in the Supplementary Figure 1 and Supplementary Table 1. The routine data was analyzed using R version 4.0.3 (22).

**TABLE 1:** Overview of study and total population.

	Total	Gender		Age			
		**Men**	**Women**	**16–24**	**25–49**	**50–64**	**65+**
	***N***	***n*** ** (Rows-%)**					
Total population Bremen 2017	584,516	286,816 (49.1)	297,700 (50.9)	71,815 (12.2)	227,428 (38.9)	141,522 (24.2)	143,751 (24.6)
Total population SHI^1^ 2017	307,245	147,025 (47.9)	160,219 (52.1)	37,706 (12.3)	117,347 (38.2)	72,263 (23.5)	79,929 (26.0)
Study population^2^ 2016/2017	11,205	7,726 (69.0)	3,479 (31.0)	577 (5.1)	3,272 (29.2)	4,448 (39.7)	2,908 (26.0)
Statutory health insurances (SHIs)	10,507	7,275 (69.2)	3,232 (30.8)	562 (5.3)	2,928 (27.9)	4,151 (39.5)	2,866 (27.3)
Gesundheit Nord – Bremen Hospital Group (GeNo)	730	503 (68.9)	227 (31.1)	19 (2.6)	345 (47.3)	302 (41.4)	64 (8.8)
German pension insurance (GPI)	343	277 (80.8)	66 (19.2)	<4	183 (53.4)	157 (45.7)	<4

^1^Including people insured in one of two statutory health insurances (SHIs) (AOK and hkk) with at least 1 day of insurance in 2017.

^2^Study population based on routine data sources (SHI, GeNo, and GPI) listed below.

#### 2.1.1. Statutory health insurance: Diagnoses

The total population of the two SHIs (AOK Bremen/Bremerhaven and hkk) consisted of insured individuals 16 years and older, living in Bremen, with at least 1 day of insurance coverage in 2016 (*N* = 302,311) or 2017 (*N* = 307,245). The population from the year 2017 was used to calculate prevalence rates of diagnoses for both years, 2016 and 2017, combined.

The ICD-10 diagnoses from services in the inpatient and outpatient setting as documented in data from the SHIs were used. All alcohol related diagnoses are presented in the Supplementary Table 1. The diagnoses E24.4, G31.2, G62.1, G72.1, I42.6, K29.2, K70.X, K86.0, O35.4, P04.3, and Q86.0 were considered other alcohol-attributable diagnoses. For a diagnosis to be counted, it had to be present at least once during the observation period. Only confirmed outpatient diagnoses or inpatient main or secondary diagnoses were used. For the extrapolation and the calculation of treatment rates, alcohol dependence was assumed if either an outpatient or an inpatient diagnosis of a dependence syndrome (F10.2) or a withdrawal state (F10.3 or 4) was coded. This approach was chosen to include individuals with only a singular diagnosis of a withdrawal state (F10.3 or 4). If both an alcohol dependence was assumed and a diagnosis of “harmful use” (F10.1) was present for an individual, only alcohol dependence was considered for the administrative prevalence.

#### 2.1.2. Statutory health insurance: Treatments

In addition to documented diagnoses in inpatient and outpatient settings, addiction-specific treatments like QWT and drug relapse prevention were also documented. QWT is a German-specific term for an extended withdrawal treatment program (generally 3 weeks) including psychosocial interventions (5). During somatic withdrawal treatment, the main focus is to control and reduce alcohol withdrawal symptoms as well as any neurological or physical symptoms (e.g., epileptic seizures or delirium tremens). In QWT, detoxification is only one component. Additionally, psychological and somatic concomitant and secondary diseases are considered and further treatment for the underlying alcohol dependence is initiated. Motivation to seek further help and more specific treatments (e.g., addiction rehabilitation) should be increased and contact should be established with the regional support system (e.g., psychotherapy, self-help) (23). The following are recommended: motivational discussion techniques; integration of family members; elements from social competence training, relaxation therapy, occupational therapy, and physiotherapy (24).

Inpatient QWT was detected using the diagnosis and the assigned Surgery- and Procedure-Code [“Operationen- und Prozeduren-Schlüssel” (OPS) code]. This official code encompasses all surgeries and medical procedures and is documented, among other reasons, for remuneration by the health insurance funds. Based on the codes, QWTs in both somatic (OPS code 8-985) and psychiatric wards (OPS code 9-647) were considered. However, these codes are not coded for specific substances. To identify a QWT for alcohol dependence, an OPS code in combination with an F10.2, 3, or 4 main diagnosis for an inpatient episode with a duration not shorter than the QWT had to be present in the observation period 2016/2017. This procedure was necessary due to partially lacking links between the OPS codes and individual inpatient episodes as well as partially missing dates for the OPS codes.

No OPS code is provided for outpatient withdrawal but, based on the pharmaceutical registration numbers [Pharmazentralnummer (PZN)], medicinal drug relapse prevention was detected. The pharmaceutical registration number and the corresponding anatomical-therapeutic-chemical classification (ATC) of the drugs prescribed and invoiced *via* the SHIs are indicative of drug relapse prevention as pharmacotherapeutic post-acute treatment in the outpatient setting (ATC code: N07BB). The assignment of the pharmaceutical registration numbers to the ATC codes was carried out based on the classification data in the drug master file of the German Drug Index of the Scientific Institute of the AOK [Wissenschaftliches Institut der AOK (WIdO)].

#### 2.1.3. Hospital group Gesundheit Nord: Outpatient addiction care

It was assumed that the data on outpatient addiction care from the GeNo represent a complete data set, as these services are free of charge and not offered based on an individual refunding system for particular services. All individuals with a documented alcohol dependence receiving outpatient addiction care from the GeNo at least once in 2016 or 2017 were included.

#### 2.1.4. German pension insurance: Rehabilitation treatment

The GPI data included individuals that at least initiated full-day outpatient or inpatient alcohol-related rehabilitation in 2016/2017 funded by the regional GPI (Deutsche Rentenversicherung Oldenburg-Bremen). As not all rehabilitation treatment is covered by the regional GPI, the total number of addiction rehabilitation cases is unknown. Besides the regional GPI there are also the federal GPI (Deutsche Rentenversicherung Bund) and GPI Knappschaft-Bahn-See (Deutsche Rentenversicherung Knappschaft-Bahn-See), which can be responsible for funding rehabilitation treatment in Bremen. Which GPI is responsible is decided for each individual by a distribution key when first being ensured in the GPI, so that 45% of insured individuals are ensured federally and 55% in one of the 16 regional GPIs depending on the place of residence. If you are employed in specific work areas, like mining sectors, German railway, and maritime shipping the GPI Knappschaft-Bahn-See is responsible. Federal GPI and GPI Knappschaft-Bahn-See together funded around 34% of the approved rehabilitation services funded overall by the GPI in the state of Bremen in 2016/2017 and the regional GPI 66% respectively (Deutsche Rentenversicherung Bund, unpublished data, 2024). Furthermore, according to the documentation of the Fachverband Sucht e.V. for 2017, the GPI (regional, federal and Knappschaft-Bahn-See) funded inpatient rehabilitation treatment in specialized clinics for alcohol and drug dependence for about 84.7% of all individuals receiving it in Germany (25). It was therefore assumed that 55.9% (0.847*0.66) of all rehabilitation services were part of the GPI data used for the extrapolation.

### 2.2. Survey data

For the overall prevalence of alcohol dependence in the population of Bremen the estimate for the whole of Germany from the 2018 Epidemiological Survey of Substance Abuse (ESA) was used. The ESA is a two-stage random sampling of the German-speaking 18–64-year-old population living in private households in Germany. The total sample was comprised of 9,287 individuals. The survey was conducted through written or online questionnaires, or telephone interviews. Alcohol dependence was determined using DSM-IV criteria (26). Details on the survey design and the methodology have been published elsewhere (3). The survey data was analyzed using Stata 15.1 (27).

### 2.3. Overall prevalence of alcohol dependence in Bremen

The ESA estimate of alcohol dependence was stratified by gender (male and female) and age groups (18–34 and 35–64 years) to account for Bremen’s unique age and gender distribution. For the population over 65 years, a logit model was calculated controlling for age (continuous), gender (male/female), education [high/medium/low according to International Standard Classification of Education (ISCED) (28)], marital status (married vs. single/divorced/widowed), and region (west/east). The probability of alcohol dependence was calculated for the group of individuals aged 65–100 years and was used as the mean prevalence for persons over 64 years (16). For the age group of 16–17-year-olds, the prevalence of 18–34-year-olds was assumed.

### 2.4. Administrative prevalence and extrapolation

First, the administrative prevalence of alcohol-related diagnoses in the routine data was calculated using the diagnoses and total population as explained in section “2.1.1. Statutory health insurance: Diagnoses.” The administrative prevalence describes the prevalence of a specific disorder in a population calculated based on routine data. Since the data sources do not share the same total population and health insurance funds record all diagnoses in inpatient and outpatient settings, the administrative prevalence was calculated based only on the population of the SHIs.

Second, extrapolations to the total population of Bremen were carried out. The overall prevalence from the ESA survey data was extrapolated to the total population of Bremen, stratified by age and gender. Population figures for Bremen in the year 2017 were taken from the Federal Statistical Office (29). As the three routine data sources cover different populations, the extrapolation of diagnoses and treatments to the federal state of Bremen was carried out for each data source (SHI, GeNo, and GPI) separately. The total population of the SHIs is shown in Table 1. For the extrapolation, the administrative prevalence, stratified by four age groups (16–24, 25–49, 50–64, and 65+ years) and by gender, was multiplied by the total population size of Bremen in 2017. When extrapolating overlapping data (hereafter: overlaps), the age and gender-stratified population not covered by the SHI data was used. This population was established by removing the total population of the SHIs’ data from the total population of Bremen. An equal distribution of diagnoses, treatments, and overlaps with the other data sets was assumed for individuals who were not included in the SHIs’ total population. These would be individuals with private insurance, another SHI, or no health insurance.

Assuming that 55.9% of all rehabilitation treatments were funded by the GPI, the extrapolated prevalence for Bremen was estimated accordingly at 614 (i.e., 343/0.559). As the gender and age distribution for the total population of persons who have undergone addiction rehabilitation treatment is unknown, the extrapolation was not stratified. The data on outpatient addiction care from the GeNo represent a complete data set, so no extrapolation had to be made.

**FIGURE 1:**
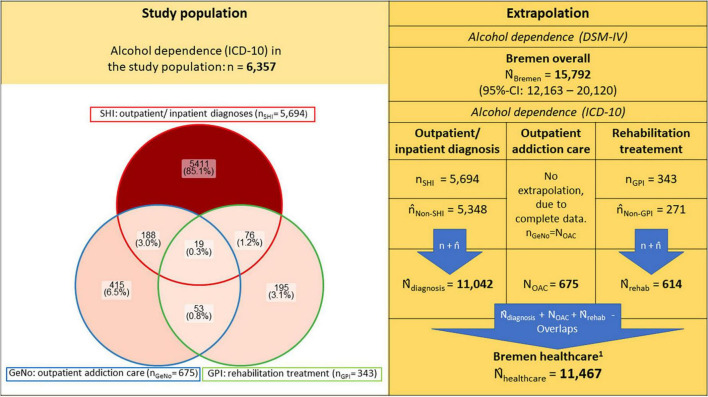
Prevalence of alcohol dependence in study population and extrapolated to total population of Bremen and its caption ^**^n/*N* denote each the empirical sample and population sizes, whereas *n̂*/*N̂* represent the estimated and extrapolated population sizes. The study population is represented as a non-proportional Venn diagram using the R package “ggVennDiagram.” For detailed extrapolations, see Supplementary Tables 2–5 and Supplementary Figure 2; SHI, Statutory health insurance; GeNo, Gesundheit Nord – Bremen Hospital Group; GPI, German Pension Insurance. ^1^Estimated individuals recognized with a diagnosis of alcohol dependence (ICD-10) or addiction specific treatment/care in the healthcare system of Bremen.

To take overlaps into account, it was assumed that the relative shares of the overlaps of persons not included in the study population correspond to those of the study population (see Figure 1). The estimated overlaps in the data sources of the unobserved populations (not in the SHI data and not in the GPI data) were calculated by multiplying the relative shares of overlaps of the observed data sources within their respective total populations by the estimated total population of each unobserved data source. Only overlaps of data from individuals covered by the observed SHIs with individuals not covered by the SHIs were stratified by age and gender in the extrapolations, as the age and gender distributions for the total population of individuals receiving rehabilitation treatment were unknown. To account for multiple counts, the overlaps were then subtracted from the sum of the extrapolations of the individual data sources again, either once (if two data sets overlapped) or twice (if three data sets overlapped).

### 2.5. Treatment rate

Specific treatment rates were estimated using the overall number of individuals with alcohol dependence in Bremen, the extrapolated administrative prevalence, and addiction-specific treatments carried out. Specific treatment rates were determined for outpatient addiction care, inpatient QWT, outpatient treatments with drug relapse prevention, and rehabilitation. No confidence intervals (CIs) were calculated for the extrapolation of treatments/care and diagnoses from the routine data. The 95% CIs shown were calculated using the respective CI limits of the overall prevalence in Bremen as the denominator for the rate.

## 3. Results

### 3.1. Administrative prevalence

The administrative prevalence of mental and behavioral disorders caused by alcohol (F10) in 2016/2017 among individuals insured by the SHIs is 2.9%, with harmful use (F10.1) at 0.8% and alcohol dependence (including withdrawal syndrome) at 1.9%. Except for “acute intoxication” and “withdrawal syndrome,” diagnoses were more often documented in outpatient than inpatient settings. Thus, the administrative prevalence of alcohol dependence (including withdrawal syndrome) in the outpatient setting is 1.6% as compared to 0.6% in the inpatient setting. The administrative prevalence of other alcohol-attributable diagnoses is 0.7% (Table 2).

**TABLE 2:** Administrative prevalence (%), stratified by diagnosis’ setting and type, in the statutory health insurances’ (SHIs) population in Bremen in 2016/2017.

ICD-10-code	Name of the diagnosis chapter	Setting and type of diagnoses				
		**Total**	**Outpatient confirmed**	**Inpatient total**	**Inpatient main**	**Inpatient secondary**
F10.X	Mental and behavioral disorders due to the use of alcohol	2.9	2.6	1.0	0.5	1.0
F10.0	Acute intoxication	0.5	0.1	0.5	0.1	0.4
F10.1	Harmful use	0.8	0.7	0.1	0.0	0.1
F10.2	Dependence syndrome	1.8	1.6	0.6	0.2	0.5
F10.3	Withdrawal state	0.4	0.1	0.3	0.2	0.2
F10.4	Withdrawal state with delirium	0.1	0.0	0.1	0.0	0.0
F10.2–4	Dependence and/or withdrawal syndrome	1.9	1.6	0.6	0.3	0.6
AAD*	Alcohol-attributable diagnoses	0.7	0.5	0.2	0.1	0.2

Inpatient total includes main or secondary diagnoses. The population includes people insured by two statutory public health insurances (AOK and hkk) with at least 1 day of insurance in 2017: *n* = 307,245.

*AAD: alcohol-attributable diagnoses include E24.4, G31.2, G62.1, G72.1, I42.6, K29.2, K70.X, K86.0, O35.4, P04.3, and Q86.0.

### 3.2. Extrapolation

Figure 1 shows the number of individuals with a documented diagnosis of alcohol dependence in the linked routine data as well as the results of the extrapolation of the overall prevalence from the ESA survey data and of each routine data set for the total population (i.e., the administrative prevalence of the SHI and the rehabilitation data). Based on survey data, the overall number of individuals with alcohol dependence in the federal state of Bremen in 2016/2017 (*N̂*_*Bremen*_) was estimated at 15,792 (95% CI: 12,163–20,120) individuals aged 16 years or older. For details see Supplementary Table 2. Based on the individuals with alcohol dependence documented in the SHI data (*n*_*SHI*_ = 5,694), extrapolated to the total population (Supplementary Table 3), we can assume an additional 5,348 individuals with alcohol dependence are documented with other health insurances or have no insurances (*n̂*_*Non–SHI*_). In addition, 675 individuals with alcohol dependence used outpatient addiction care services (n_*GeNo*_). When extrapolated to the total population, we estimate 614 individuals made use of addiction rehabilitation (*n*_*GPI*_ = 343; *n̂*_*Non–GPI*_ = 271). The results of the extrapolation of the overlaps between the data sources to adjust for multiple counts is presented in Supplementary Figure 2 and the extrapolations are shown in Supplementary Tables 4, 5. The number of individuals with alcohol dependence documented in the health system was estimated at 11,467 (*n*_*SHI*_ + *n̂*_*Non–SHI*_ + *n*_*GeNo*_ + *n*_*GPI*_ + *n̂*_*Non–GPI*_–Overlaps). All extrapolations are shown in detail in the Supplementary Figure 2 and Supplementary Tables 2–5.

### 3.3. Addiction-specific treatments and care

**FIGURE 2:**
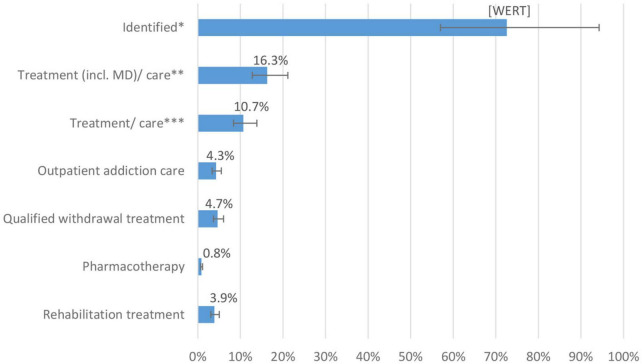
Diagnoses and specific treatment/care rates of persons with alcohol dependence in Bremen 2016/2017 and its caption ^**^Proportions of extrapolated treatments in the estimate for persons with alcohol dependence in the total population of Bremen *N̂*_*Bremen*_ 15,792 [12,163–20,120]: *Identified includes individuals with at least one outpatient or inpatient diagnosis, utilization of outpatient addiction care or addiction rehabilitation ^**^Treatment (incl. MD)/care include here: inpatient episode with main diagnosis F10.2–4, qualified withdrawal treatment, pharmacotherapy, outpatient addiction care, and rehabilitation treatment ^***^Treatment/care include here: qualified withdrawal treatment, pharmacotherapy, outpatient addiction care, and rehabilitation treatment.

The extrapolated general and specific treatment rates for individuals with alcohol dependence in the total population are shown in Figure 2. Overall, 72.6% [95% CI: 57.8%–94.3%] of the estimated total number of individuals with alcohol dependence and a corresponding ICD-10 diagnosis were registered in the health care system. For 61.9%, no addiction-specific treatments were identified. The share of individuals with at least one of the treatments or care measures considered here was 10.7% [95% CI: 8.4%–13.9%]. Based on the estimate of the overall prevalence and the extrapolation of the routine data, inpatient QWT was initiated by 4.7% [95% CI: 3.7%–6.1%], whereas 4.3% [95% CI: 3.4%–5.5%] used outpatient addiction care services, 3.9% [95% CI: 3.1%–5.0%] used addiction rehabilitation, and 0.8% [95% CI: 0.7%–1.1%] used outpatient drug-based relapse prevention interventions (i.e., anticraving medications).

**FIGURE 3:**
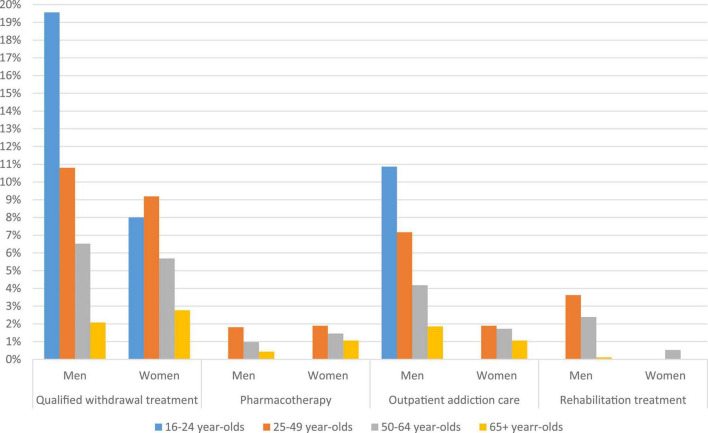
Specific treatment/care rates of the statutory health insurances’ (SHIs) population with an alcohol dependence syndrome diagnosis (F10.2–4) in 2016/2017, stratified by age and gender. SHIs’ population with F10.2–4 diagnosis in 2016/2017: 16–24-year-olds (*n*_*M*_ = 46; *n*_*W*_ = 25); 25–49-year-olds (*n*_*M*_ = 1,269; *n*_*W*_ = 370); 50–64-year-olds (*n*_*M*_ = 1,841; *n*_*W*_ = 756); and over 64-year-olds (*n*_*M*_ = 917; *n*_*W*_ = 470).

Figure 3 shows the proportion of individuals with a diagnosed alcohol dependence in the SHIs’ population who used specific treatment/care, stratified by age and gender with a diagnosed alcohol dependence. Overall, the shares of individuals in treatment were higher in younger than older individuals. Gender differences were observed in QWT, outpatient addiction care, and rehabilitation treatment. Compared to young women up to age 24, young men with alcohol dependence more often started QWT (19.6% vs. 8.0%) or attended outpatient addiction care (10.9% vs. 0.0%). For rehabilitation treatment (3.6% vs. 0.0%) and outpatient addiction care (7.2% vs. 1.9%), gender differences were also present in individuals aged 25–49 years.

**FIGURE 4:**
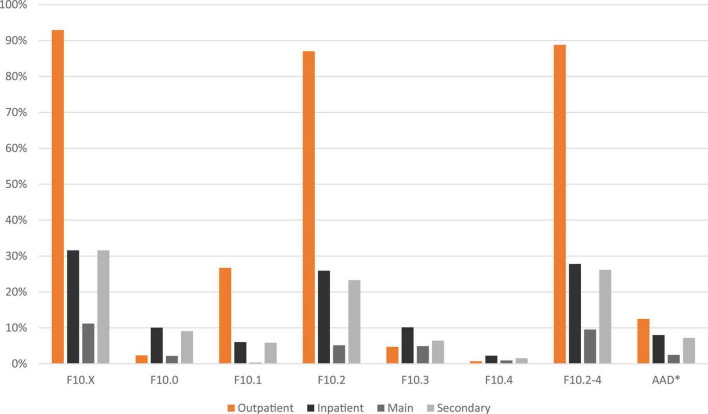
Diagnosis and diagnosis setting of persons with an alcohol dependence syndrome diagnosis (F10.2–4) but without addiction-specific care in the statutory health insurances’ (SHIs) population in 2016/2017. Outpatient (confirmed diagnosis only), inpatient (main or secondary diagnosis); the proportion of individuals in statutory public health insurances’ population with a diagnosis of F10.2–4 but without addiction-specific care: *n* = 5,097. *AAD: alcohol-attributable diagnoses include E24.4, G31.2, G62.1, G72.1, I42.6, K29.2, K70.X, K86.0, O35.4, P04.3, and Q86.0.

Figure 4 shows the prevalence of F10 disorders and the diagnosis setting for individuals in the SHIs’ population with a diagnosis but without identified treatment/care. Most of these individuals received an outpatient diagnosis for alcohol dependence (88.8%) and significantly fewer receive an inpatient diagnosis (27.8%) mainly as a secondary diagnosis. Comparable to the general administrative prevalence of F10 disorders only diagnoses of “acute intoxication” (F10.0) and “withdrawal state” (F10.3 and 4) were documented more often in inpatient than in outpatient settings.

## 4. Discussion

The number of individuals with alcohol dependence in the Federal State of Bremen in 2017 was estimated at 15,792 [95% CI: 12,163–20,120]. Of these, 11,467 persons (73% [95% CI: 57%–94%]) received a corresponding ICD diagnosis in medical health care or outpatient addiction care in 2016/2017 and 11% [95% CI: 8%–14%] made use of addiction-specific care measures according to our estimates. Despite limited comparability due to a longer observation period and different data sources, the treatment rates largely correspond to the Germany-wide estimate from 2012, although a higher diagnosis rate seems to be present in Bremen (16).

Previous routine data analyses showed high prevalence rates of mental disorders in outpatient care, especially in general medicine, but a relatively low proportion in psychiatric or psychotherapeutic care (30). Survey data confirm general practitioners as the most frequently visited contact person and the first point of access to the health care system for alcohol-related problems (31). Consistently, most diagnoses in Bremen were made on an outpatient basis. This emphasizes the importance of primary health care for the identification and further treatment of alcohol dependence, especially as individuals who do not make use of further addiction-specific care very often only receive an outpatient diagnosis. Whether any treatments took place in this setting in addition to the pharmacotherapy shown and, if so, which ones, could not be determined. In general, an increase in addiction-specific treatment rates seems to be indicated due to possible positive effects on per capita alcohol consumption (32), mortality (33, 34), and hospitalization rates (35).

However, the figures reported here do not reflect the actual treatment gap, as not all individuals with alcohol dependence are actually in need of addiction-specific treatment. A study from the Netherlands estimated the treatment gap for individuals with alcohol use disorder according to the DSM-V to be significantly lower if only individuals with persistent alcohol use disorders (over 4 years) were considered (24.5% instead of 90.0%) (36). Treatment gap estimates need to account for remissions without formal help, which depend on the time interval and the definition of both treatment and remission (37, 38). Previous estimates of natural remissions in individuals with alcohol dependence are about three quarters of cases in Northern America (37, 39) and 66% in Germany (40).

The overall low utilization of addiction-specific treatments and simultaneously high diagnosis rate of alcohol dependence indicate that the care of patients after diagnosis is challenging and tedious. Previous research identified various treatment provision barriers for general practitioners in Germany. For example, referral to the addiction support system is limited among other reasons due to a lack of networking (8). The often uncoordinated access to various care systems sometimes results in a lack of exchange of relevant information (41). A lack of health policy and financial support, as well as a lack of time, are often reported as reasons for the low use of brief interventions and screenings (8, 11, 42, 43). Further barriers to treatment provision include a general lack of private practice physicians that are qualified to adequately treat patients with alcohol dependence as well as physicians’ negative expectations of patient adherence (8, 11, 12, 43). On the patient side, a lack of self-awareness, the desire to keep drinking, and the fear of stigmatization or shame were shown to be the most important reasons against seeking further treatment (13, 43). Compared to other mental illnesses, the stigmatization of alcohol use disorders is particularly pronounced (14). Impacts thereof can be seen in the late use of cessation treatments, as, on average, it had been almost 16 years since the onset of symptoms for patients in addiction-specific care (44).

In this study, the proportion of diagnosed individuals with addiction-specific care was lower in older age groups. Although fewer individuals were diagnosed in absolute terms in the younger age groups, the treatment rate was higher among them. An inverted U-shaped relationship with the peak treatment rate occurring in middle age (between 35 and 54 years), as in the USA, is not evident in the already diagnosed population. Rather there is a linear decrease (45). The higher rate of care among 16–24-year-olds may be due to covariates such as comorbidities and risky drinking habits, as well as a presumably lower rate of diagnosis among younger individuals with alcohol dependence. In general, older patients with comorbidities are more likely to be diagnosed (19) and treatment motivation increases with the severity of negative consequences (45, 46).

The lower treatment rates among women found in our study are in line with the current literature (47–49). A literature review identified a lower perceived need for treatment in women compared to men as well as more guilt and shame, less social support, and different socioeconomic characteristics and comorbidities (49). Furthermore, women tend to seek care more often in non-substance abuse-specific settings (47). These important covariables were not controlled for in our study, making a gender specific analysis difficult. In this study, differences in treatment utilization were apparent, but not for all treatments. Differences in QWT were only seen in the youngest age group and could be due to the low absolute number of women in this age group in the study population. The lower utilization rate of outpatient addiction care and rehabilitation treatment for women as compared to men may also indicate that women utilize treatment approaches other than addiction care and rehabilitation.

### 4.1. Strengths and limitations

The strength of the routine data used here lies in the fact that the utilization of individual addiction-specific measures can be recorded validly without typical survey biases such as memory errors, non-response, or social desirability bias. In contrast to the use of aggregated data, the data linkage procedure made it possible to assign the respective services used to each individual. Generally, the highly fragmented care system for individuals with alcohol use disorders with many potential payers complicates a complete view of this population in Germany.

The available routine data is limited to medical care and extrapolations are based on the assumption of an equal distribution of certain characteristics across data sets from different payers. The assumption of equal proportions of QWT in individuals with statutory versus private and non-SHI is only valid to a limited extent. Not all private insurances cover the costs of QWT, and those who are not insured are unlikely to always be able to finance it. In addition, QWT was coded based on the start of treatment, and thus our data represent initiated but not necessarily completed courses of treatment. Likewise, outpatient withdrawal treatment, which is only possible when patients meet certain treatment criteria, was not considered. The implementation of physical detoxification could not be presented as a specific treatment rate, as this is not specifically documented (e.g., by means of an OPS code). However, if inpatient main diagnoses of alcohol dependence were considered in calculating the general treatment rate, not only qualified withdrawal but also inpatient physical detoxification would be included (treatment rate with and without inpatient main diagnosis as a treatment: 16.3% vs. 10.7%). Pharmacotherapy was considered independently of previous measures, because it seems reasonable to assume that treatment is carried out after withdrawal or rehabilitation treatment and thus as post-acute treatment.

The routine data analyzed here mainly show the reimbursed services and, to a limited extent, the periods of illness. This should be taken into consideration, especially given that alcohol dependence is a chronic disease. It should be emphasized that the measures reflected by the available data are not exhaustive. Brief interventions, medical consultations, or outpatient psychotherapeutic measures were not documented in the available routine data. It was also not possible to consider self-help and counseling services outside the GeNo due to a lack of available documentation.

A prevalence estimate from a general population survey was used to estimate the number of individuals with alcohol dependence in Bremen. The influence of non-response and groups not included in the study, such as prison inmates and homeless people with a higher risk of alcohol dependence, indicate this is a conservative estimate (3). In addition, the use of the estimated Germany-wide prevalence of alcohol dependence as a proxy for the federal state of Bremen does not consider regional differences. The state of Bremen is located in Northwest Germany and, compared to the German average, Bremen has a higher share of migrants (19% vs. 13%) (50), is younger (mean age 43.7 vs. 44.6) (51), has the highest share of people near poverty (52), and a higher rate of alcohol-attributable mortality (53, 54). Thus, the overall prevalence of alcohol dependence in Bremen is likely underestimated. Treatment and diagnosis rates, therefore, tend to be overestimated.

Differences between individuals diagnosed by primary health care, using parts of the routine data, and utilizing clinical diagnostic interviews, which were used for the estimation of the total population of individuals with alcohol dependence in Germany, are irrelevant for an estimation of the total population. In a Europe-wide study, general practitioners were more likely to diagnose alcohol dependence in older, male persons with more somatic comorbidities in comparison to standardized diagnostic interviews, but a similar number of diagnoses were made using either diagnostic method (19).

## 5. Conclusion

The analyzed secondary data shows a clear picture of the care and treatment that individuals with alcohol dependence receive. The results point to a discrepancy between outpatient diagnosis and the utilization of addiction-specific treatment services. More than half of the individuals with alcohol dependence in Bremen were identified in the health care system, but only a minority of them received addiction-specific treatment. Despite a broad consensus and an existing guideline in Germany with measures for a stronger networking between care sectors and seamless access to addiction-specific measures, the barriers are still considerable in practice. Ideally, addiction-specific measures should be initiated at an early stage so that treatment is not forced only by existing negative consequences. This also requires a greater self-awareness among this population to increase the patients’ motivation for receiving treatment. Improving how primary care providers treat individuals with alcohol-related disorders, as well as increasing networking within the addiction care system, seems particularly appropriate.

## Data availability statement

The data analyzed in this study is subject to the following licenses/restrictions: The data holders restrict the analysis of the IMPELA dataset to the applicants of the project for commercial in confidence reasons. Requests to access these datasets should be directed to https://www.impela.de/kontakt/.

## Author contributions

LK, CL, JaM, BS, and JR designed the study. JuM analyzed the data and wrote the initial draft of the manuscript. LK, JaM, CL, and BS gave important feedback on the analysis and the interpretation of the results. All authors commented on various versions of the manuscript and approved the final version.

## References

[1] John U Hanke M Freyer-Adam J Baumann S Meyer C. Alkohol. In: Deutsche Hauptstelle für Suchtfragen DHS editor. *Jahrbuch Sucht*. Lengerich: Papst Sience Publisher (2021). p. 37–54.

[2] World Health Organization. *Recorded Alcohol per Capita Consumption, from 2010.* Geneva: World Health Organization (2021).

[3] Atzendorf J Rauschert C Seitz N Lochbuhler K Kraus L. The use of alcohol, tobacco, illegal drugs and medicines: an estimate of consumption and substance-related disorders in Germany. *Dtsch Arztebl Int.* (2019) 116:577–84. 10.3238/arztebl.2019.0577 31587705 PMC6804269

[4] Bölt U. Statistische krankenhausdaten: grunddaten der krankenhäuser 2018. In: Klauber J Wasem J Beivers A Mostert C editors. *Krankenhaus-Report 2021: Versorgungsketten – Der Patient im Mittelpunkt.* (Berlin: Springer) (2021). p. 375–403. 10.1007/978-3-662-62708-2_19

[5] Kiefer F Batra A Petersen K Ardern I Tananska D Bischof G German guidelines on screening, diagnosis, and treatment of alcohol use disorders: update 2021. *Eur Addict Res.* (2022) 28:309–22. 10.1159/000522335 35439764

[6] Trautmann S Pieper L Kuitunen-Paul S Manthey J Wittchen H Bühringer G Prävalenz und behandlungsraten von störungen durch alkoholkonsum in der primärärztlichen versorgung in Deutschland. *Sucht.* (2016) 62:233–43. 10.1024/0939-5911/a000434

[7] Röhrig J Flaig S Niebling W Ruf D Wahl S Berner M. Früherkennung und behandlung alkoholbezogener störungen: eine prä-post-studie zur verbesserung der vernetzung von hausarzt und suchtberatung. *Suchttherapie.* (2011) 12:134–40. 10.1055/s-0031-1284361

[8] Schulte B Schmidt C Milin S Farnbacher G Schäfer I Bleich S Barrieren und möglichkeiten in der umsetzung von alkoholbezogenen interventionen in der hausärztlichen praxis. *Suchttherapie.* (2014) 15:35–42. 10.1055/s-0033-1349863

[9] Mekonen T Chan G Connor J Hall W Hides L Leung J. Treatment rates for alcohol use disorders: a systematic review and meta-analysis. *Addiction.* (2020) 116:2617–34. 10.1111/add.15357 33245581

[10] Kohn R Saxena S Levav I Saraceno B. The treatment gap in mental health care. *Bull World Health Organ.* (2004) 82:858–66. 15640922 PMC2623050

[11] Fankhaenel T Klement A Forschner L. Hausärztliche intervention für eine entwöhnungs- langzeitbehandlung bei patienten mit einer suchterkrankung (HELPS). *Sucht Aktuell.* (2014) 2:55–9.

[12] Hoffmann T Voigt K Kugler J Peschel L Bergmann A Riemenschneider H. Are German family practitioners and psychiatrists sufficiently trained to diagnose and treat patients with alcohol problems? *BMC Fam Pract.* (2019) 20:115. 10.1186/s12875-019-1006-8 31416419 PMC6694527

[13] Probst C Manthey J Martinez A Rehm J. Alcohol use disorder severity and reported reasons not to seek treatment: a cross-sectional study in European primary care practices. *Subst Abuse Treat Prev Policy.* (2015) 10:32. 10.1186/s13011-015-0028-z 26264215 PMC4534056

[14] Kilian C Manthey J Carr S Hanschmidt F Rehm J Speerforck S Stigmatization of people with alcohol use disorders: an updated systematic review of population studies. *Alcohol Clin Exp Res.* (2021) 45:899–911. 10.1111/acer.14598 33970504

[15] Rehm J Allamani A Elekes Z Jakubczyk A Manthey J Probst C Alcohol dependence and treatment utilization in Europe - a representative cross-sectional study in primary care. *BMC Fam Pract.* (2015) 16:90. 10.1186/s12875-015-0308-8 26219430 PMC4518612

[16] Kraus L Piontek D Pfeiffer-Gerschel T Rehm J. Inanspruchnahme gesundheitlicher versorgung durch alkoholabhängige. *Suchttherapie.* (2015) 16:18–26. 10.1055/s-0034-1376999

[17] Manthey J Lindemann C Verthein U Frischknecht U Kraus L Reimer J Versorgung von personen mit riskantem alkoholkonsum und schwerer alkoholkonsumstörung in Bremen: bedarfsgerecht und leitlinienkonform? *Bundesgesundheitsblatt Gesundheitsforschung Gesundheitsschutz.* (2020) 63:122–30. 10.1007/s00103-019-03072-z 31828370

[18] Kuitunen-Paul S Manthey J Trautmann S Pieper L Wittchen H Rehm J. Alkoholabhängigkeit in der primärärztlichen Versorgung: welche Patienten werden erkannt? *Suchttherapie.* (2016) 18:82–9. 10.1055/s-0042-113143

[19] Rehm J Manthey J Struzzo P Gual A Wojnar M. Who receives treatment for alcohol use disorders in the European Union? A cross-sectional representative study in primary and specialized health care. *Eur Psychiatry.* (2015) 30:885–93. 10.1016/j.eurpsy.2015.07.012 26647862

[20] Schulte B Lindemann C Buchholz A Rosahl A Härter M Kraus L Tailored interventions to support the implementation of the German national guideline on screening, diagnosis and treatment of alcohol-related disorders: a project protocol. *Sucht.* (2019) 65:373–81. 10.1024/0939-5911/a000629

[21] BMG. *Daten des Gesundheitswesens 2021.* Berlin: Bundesministerium für Gesundheit (2021).

[22] R Core Team. *R: A Language and Environment for Statistical Computing.* Vienna: R Foundation for Statistical Computing (2021).

[23] Mann K Batra A Fauth-Bühler M Hoch E. German guidelines on screening, diagnosis and treatment of alcohol use disorders. *Eur Addict Res.* (2017) 23:45–60. 10.1159/000455841 28178695

[24] Batra A Muller C Mann K Heinz A. Alcohol dependence and harmful use of alcohol. *Dtsch Arztebl Int.* (2016) 113:301–10. 10.3238/arztebl.2016.0301 27173413 PMC4873678

[25] Bachmeier R Bick-Dresen S Dreckmann I Holger F Kemmann D Kersting S *Basisdokumentation 2017 – Fachkliniken für Alkohol-, Medikamentenabhängigkeit.* Bonn: Fachverband Sucht e. V (2018).

[26] Lachner G Wittchen H Perkonigg A Holly A Schuster P Wunderlich U Structure, content and reliability of the munich-composite international diagnostic interview (M-CIDI) substance use sections. *Eur Addict Res.* (1998) 4:28–41. 10.1159/000018922 9740815

[27] StataCorp. *Stata Statistical Software: Release 15.* College Station, TX: StataCorp LLC (2017).

[28] Unesco. *International Standard Classification of Education ISCED 2011.* Montreal: UNESCO Institute for Statistics (2012).

[29] Destatis. *Total Population of Bremen and by Age and Gender 2017.* Wiesbaden: Statistisches Bundesamt (2022).

[30] Gaebel W Kowitz S Fritze J Zielasek J. Inanspruchnahme des versorgungssystems bei psychischen Erkrankungen. *Dtsch Arztebl Int.* (2013) 110:799–808. 24314623 10.3238/arztebl.2013.0799 PMC3859909

[31] Gomes de Matos E Kraus L Pabst A Piontek D. roblembewusstsein und Inanspruchnahme von Hilfe bei substanzbezogenen Problemen. *Sucht.* (2013) 59:355–66. 10.1024/0939-5911.a000278

[32] Manthey J Solovei A Anderson P Carr S Rehm J. Can alcohol consumption in Germany be reduced by alcohol screening, brief intervention and referral to treatment in primary health care? Results of a simulation study. *PLoS One.* (2021) 16:e0255843. 10.1371/journal.pone.0255843 34352005 PMC8341530

[33] Rehm J Rehm M Shield K Gmel G Frick U Mann K. Reduzierung alkoholbedingter mortalität durch behandlung der alkoholabhängigkeit. *Sucht.* (2014) 60:93–105. 10.1024/0939-5911.a000299

[34] Manthey J Lindemann C Kraus L Reimer J Verthein U Schulte B The potential effects of an extended alcohol withdrawal treatment programme on morbidity and mortality among inpatients in the German city of Bremen: a simulation study. *Subst Abuse Treat Prev Policy.* (2020) 15:1. 10.1186/s13011-019-0249-7 31898529 PMC6941395

[35] Heikkinen M Taipale H Tanskanen A Mittendorfer-Rutz E Lähteenvuo M Tiihonen J. Real-world effectiveness of pharmacological treatments of alcohol use disorders in a Swedish nation-wide cohort of 125?556 patients. *Addiction.* (2021) 116:1990–8. 10.1111/add.15384 33394527 PMC8359433

[36] Tuithof M ten Have M van den Brink W Vollebergh W de Graaf R. Treatment seeking for alcohol use disorders: treatment gap or adequate self-selection? *Eur Addict Res.* (2016) 22:277–85. 10.1159/000446822 27287873

[37] Rumpf H Bischof G Hapke U Meyer C John U. Remission ohne formelle Hilfe bei Alkoholabhängigkeit: Der Stand der Forschung. *SUCHT.* (2009) 55:75–85. 10.1024/2009.02.03

[38] Ritter A Mellor R Chalmers J Sunderland M Lancaster K. Key considerations in planning for substance use treatment: estimating treatment need and demand. *J Stud Alcohol Drugs Suppl.* (2019) 18:22–30. 10.15288/jsads.2019.s18.22 30681945 PMC6377022

[39] Dawson D. Correlates of past-year status among treated and untreated persons with former alcohol dependence: United States, 1992. *Alcohol Clin Exp Res.* (1996) 20:771–9. 10.1111/j.1530-0277.1996.tb01685.x 8800398

[40] Rumpf H Meyer C Hapke U Bischof G John U. Inanspruchnahme suchtspezifischer Hilfen von Alkoholabhängigen und -mißbrauchern: ergebnisse der TACOS-Bevölkerungsstudie. *Sucht.* (2000) 46:9–17. 10.1024/suc.2000.46.1.9

[41] Penm J MacKinnon N Strakowski S Ying J Doty M. Minding the gap: factors associated with primary care coordination of adults in 11 countries. *Ann Fam Med.* (2017) 15:113–9. 10.1370/afm.2028 28289109 PMC5348227

[42] Kraus L Schulte B Manthey J Rehm J. Alcohol screening and alcohol interventions among patients with hypertension in primary health care: an empirical survey of German general practitioners. *Addict Res Theory.* (2017) 25:285–92. 10.1080/16066359.2016.1263728

[43] Buchholz A Spies M Härter M Lindemann C Schulte B Kiefer F Barrieren und Umsetzungsstrategien für die Implementierung der S3-Leitlinie Screening, Diagnose und Behandlung alkoholbezogener Störungen aus Sicht von Behandlern und Betroffenen. *Suchttherapie.* (2021) 66–76. 10.1055/a-1324-5217

[44] Bachmeier R Bick-Dresen S Funke W Kemmann D Kersting S Klein T *Basisdokumentation 2019 – Fachkliniken für Alkohol-, Medikamentenabhängigkeit*. Bonn: Fachverband Sucht e. V (2020).

[45] Venegas A Donato S Meredith L Ray L. Understanding low treatment seeking rates for alcohol use disorder: a narrative review of the literature and opportunities for improvement. *Am J Drug Alcohol Abuse.* (2021) 47:664–79. 10.1080/00952990.2021.1969658 34464542 PMC9059657

[46] Rohn M Lee M Kleuter S Schwandt M Falk D Leggio L. Differences between treatment-seeking and nontreatment-seeking alcohol-dependent research participants: an exploratory analysis. *Alcohol Clin Exp Res.* (2017) 41:414–20. 10.1111/acer.13304 28129451 PMC6468994

[47] McCaul M Roach D Hasin D Weisner C Chang G Sinha R. Alcohol and women: a brief overview. *Alcohol Clin Exp Res.* (2019) 43:774–9. 10.1111/acer.13985 30779446 PMC6502688

[48] Gilbert P Pro G Zemore S Mulia N Brown G. Gender differences in use of alcohol treatment services and reasons for nonuse in a national sample. *Alcohol Clin Exp Res.* (2019) 43:722–31. 10.1111/acer.13965 30807660 PMC6443428

[49] Maxwell A Harrison K Rawls E Zilverstand A. Gender Differences in the psychosocial determinants underlying the onset and maintenance of alcohol use disorder. *Front Neurosci.* (2022) 16:808776. 10.3389/fnins.2022.808776 35360152 PMC8964095

[50] DESTATIS. *Bevölkerung am 31.12.2021 Nach Nationalität und Bundesländern.* Wiesbaden: Statistisches Bundesamt (2022).

[51] DESTATIS. *Bevölkerung und Erwerbstätigkeit. Bevölkerungsfortschreibung auf Grundlage des Zensus 2011. 1.* 3 ed. Wiesbaden: Statistisches Bundesamt (2020).

[52] DESTATIS. *Armutsgefährdung in Bremen, Hessen und Nordrhein-Westfalen von 2009 bis 2019 am stärksten gestiegen.* Wiesbaden: Statistisches Bundesamt (2020).

[53] Rommel A Saß A Rabenberg M. Alkoholbedingte mortalität bei erwachsenen. *J Health Monit*. (2016) 1:37–42. 10.17886/RKI-GBE-2016-022

[54] Kilian C Carr S Schulte B Manthey J. Increased alcohol-specific mortality in Germany during COVID-19: state-level trends from 2010 to 2020. *Drug Alcohol Rev.* (2022). 10.1111/dar.13573 36352737 PMC9878067

