# Corrigendum: Estimating the prevalence of alcohol-related disorders and treatment utilization in Bremen 2016/2017 through routine data linkage

**DOI:** 10.3389/fpsyt.2024.1393643

**Published:** 2024-05-27

**Authors:** Justin Möckl, Christina Lindemann, Jakob Manthey, Bernd Schulte, Jens Reimer, Oliver Pogarell, Ludwig Kraus

**Affiliations:** ^1^Department of Epidemiology and Diagnostics, Institut für Therapieforschung (IFT), Centre for Mental Health and Addiction Research, Munich, Germany; ^2^Department of Psychiatry and Psychotherapy, University Hospital, Ludwig-Maximilians-Universität Munich, Munich, Germany; ^3^Department of Psychiatry and Psychotherapy, Center for Interdisciplinary Addiction Research, University Medical Center Hamburg-Eppendorf, Hamburg, Germany; ^4^Department of Medical Psychology, Center for Health Care Research, University Medical Center Hamburg-Eppendorf, Hamburg, Germany; ^5^Department of Psychiatry, Medical Faculty, University of Leipzig, Leipzig, Germany; ^6^Zentrum für Psychosoziale Medizin, Klinikum Itzehoe, Itzehoe, Germany; ^7^Department of Public Health Sciences, Centre for Social Research on Alcohol and Drugs, Stockholm University, Stockholm, Sweden; ^8^Institute of Psychology, Eötvös Loránd University (ELTE), Budapest, Hungary

**Keywords:** alcohol dependence, treatment utilization, data linkage, routine data, epidemiology

In the published article, there was an error concerning the assumptions of the extrapolation. It was mistakenly assumed that the data of the German pension insurance (Deutsche Rentenversicherung, DRV) covers all rehabilitations in Bremen funded by the German pension insurance. However, the data only contains rehabilitation treatments covered by the regional German pension insurance (DRV Oldenburg-Bremen) and rehabilitation treatments can also be funded by the federal German pension insurance (DRV Bund) and German pension insurance Knappschaft-Bahn-See (DRV Knappschaft-Bahn-See). This was erroneously not considered in the extrapolation. This error was now fixed, and the extrapolation was corrected, which lead to several corrections throughout the manuscript and Supplementary material.

A correction has been made to Abstract, *Results*. This paragraph previously stated:

“Of the survey-estimated 15,792 individuals with alcohol dependence in Bremen, 72.4% (*n* = 11,427) had a diagnosis documented with an ICD-10 code for alcohol dependence (F10.2) or withdrawal state (F10.3–4). One in 10 individuals with alcohol dependence (*n* = 1,577) used one or more addiction-specific care services during the observation period. Specifically, 3.7% (*n* = 675) received outpatient addiction care, 3.9% (*n* = 736) initiated QWT, 0.8% (*n* = 133) received pharmacotherapy, and 2.6% (*n* = 405) underwent rehabilitation treatment. The share of seeking addiction-specific treatment after diagnosis was highest among younger and male patients.”

The corrected paragraph appears below:

“Of the survey-estimated 15,792 individuals with alcohol dependence in Bremen, 72.6% (*n* = 11,467) had a diagnosis documented with an ICD-10 code for alcohol dependence (F10.2) or withdrawal symptoms (F10.3–F10.4). One in ten individuals with alcohol dependence (*n* = 1,689) used one or more addiction-specific care services during the observation period. Specifically, 4.3% (*n* = 675) received outpatient addiction care, 4.7% (*n* = 736) initiated QWT, 0.8% (*n* = 133) received pharmacotherapy, and 3.9% (*n* = 614) underwent rehabilitation treatment. The share of seeking addiction-specific treatment after diagnosis was highest among younger and male patients.”

A correction has been made to 2. Materials and methods, *2.1. Study population*, paragraph 2. This sentence previously stated:

“To this end, regional master data and service data from 2016 and 2017 from (1) two SHIs in Bremen (AOK Bremen/Bremerhaven and hkk), (2) on outpatient addiction care services data of the communal hospital group Gesundheit Nord – Bremen Hospital Group (GeNo) in Bremen, and (3) the GPI were linked on an individual level (20).”

The corrected sentence appears below:

“To this end, regional master data and service data from 2016 and 2017 from (1) two SHIs in Bremen (AOK Bremen/Bremerhaven and hkk), (2) on outpatient addiction care services data of the communal hospital group Gesundheit Nord–Bremen Hospital Group (GeNo) in Bremen, and (3) the regional GPI (Deutsche Rentenversicherung Oldenburg-Bremen) were linked on an individual level (20).”

A correction has been made to 2. Materials and methods, *2.1.4 German pension insurance: Rehabilitation treatment*, paragraph 1. This paragraph previously stated:

“The GPI data included individuals that at least initiated full-day outpatient or inpatient alcohol-related rehabilitation in 2016/2017. As not all rehabilitation treatment is covered by the GPI, the total number of addiction rehabilitation cases is unknown. According to the documentation of the Fachverband Sucht e.V. for 2017, the GPI funded inpatient rehabilitation treatment in specialized clinics for alcohol and drug dependence for about 84.7% of all individuals receiving it in Germany (25).”

The corrected sentence appears below:

“The GPI data included individuals that at least initiated full-day outpatient or inpatient alcohol-related rehabilitation in 2016/2017 funded by the regional GPI (Deutsche Rentenversicherung Oldenburg-Bremen). As not all rehabilitation treatment is covered by the regional GPI, the total number of addiction rehabilitation cases is unknown. Besides the regional GPI there are also the federal GPI (Deutsche Rentenversicherung Bund) and GPI Knappschaft-Bahn-See (Deutsche Rentenversicherung Knappschaft-Bahn-See), which can be responsible for funding rehabilitation treatment in Bremen. Which GPI is responsible is decided for each individual by a distribution key when first being ensured in the GPI, so that 45% of insured individuals are ensured federally and 55% in one of the 16 regional GPIs depending on the place of residence. If you are employed in specific work areas, like mining sectors, German railway, and maritime shipping the GPI Knappschaft-Bahn-See is responsible. Federal GPI and GPI Knappschaft-Bahn-See together funded around 34% of the approved rehabilitation services funded overall by the GPI in the state of Bremen in 2016/2017 and the regional GPI 66% respectively (Deutsche Rentenversicherung Bund, unpublished data, 2024). Furthermore, according to the documentation of the Fachverband Sucht e.V. for 2017, the GPI (regional, federal and Knappschaft-Bahn-See) funded inpatient rehabilitation treatment in specialized clinics for alcohol and drug dependence for about 84.7% of all individuals receiving it in Germany (25). It was therefore assumed that 55.9% (0.847^*^0.66) of all rehabilitation services were part of the GPI data used for the extrapolation.”

A correction has been made to 2. Materials and methods, *2.4. Administrative prevalence and extrapolation*, paragraph 3. This sentence previously stated:

“Assuming that 84.7% of all rehabilitation treatments were funded by the GPI, the extrapolated prevalence for Bremen was estimated accordingly at 405 (i.e., 343/0.847).”

The corrected sentence appears below:

“Assuming that 55.9% of all rehabilitation treatments were funded by the GPI, the extrapolated prevalence for Bremen was estimated accordingly at 614 (i.e., 343/0.559).”

A correction has been made to 3. Results, *3.2. Extrapolation*, Paragraph 1. This paragraph previously stated:

“When extrapolated to the total population, we estimate 405 individuals made use of addiction rehabilitation (nGPI = 343; nNon–GPI = 62). The results of the extrapolation of the overlaps between the data sources to adjust for multiple counts is presented in Supplementary Figure 2 and the extrapolations are shown in Supplementary Tables 4, 5. The number of individuals with alcohol dependence documented in the health system was estimated at 11,427 (nSHI + nNon–SHI + nGeNo + nGPI + nNon–GPI–Overlaps). All extrapolations are shown in detail in the Supplementary Figure 2 and Supplementary Tables 2–5.”

The corrected paragraph appears below:

“When extrapolated to the total population, we estimate 614 individuals made use of addiction rehabilitation (nGPI = 343; nNon-GPI = 271). The results of the extrapolation of the overlaps between the data sources to adjust for multiple counts is presented in Supplementary Figure 2 and the extrapolations are shown in Supplementary Tables 4, 5. The number of individuals with alcohol dependence documented in the health system was estimated at 11,467 (nSHI + nNon-SHI + nGeNo + nGPI + nNon-GPI – Overlaps). All extrapolations are shown in detail in the Supplementary Figure 2 and Supplementary Tables 2–5.”

A correction has been made to 3. Results, *3.3. Addiction-specific treatments and care*, first Paragraph. This sentence previously stated:

“The extrapolated general and specific treatment rates for individuals with alcohol dependence in the total population are shown in Figure 2. Overall, 72.4% (95% CI: 56.8–93.3%) of the estimated total number of individuals with alcohol dependence and a corresponding ICD-10 diagnosis were registered in the health care system. For 62.4%, no addiction-specific treatments were identified. The share of individuals with at least one of the treatments or care measures considered here was 10.0% (95% CI: 7.8–13.0%). Based on the estimate of the overall prevalence and the extrapolation of the routine data, inpatient QWT was initiated by 4.7% (95% CI: 3.7–6.1%), whereas 4.3% (95% CI: 3.4–5.5%) used outpatient addiction care services, 2.6% (95% CI: 2.0–3.3%) used addiction rehabilitation, and 0.8% (95% CI: 0.7–1.1%) used outpatient drug-based relapse prevention interventions (i.e., anti-craving medications).”

The corrected sentence appears below:

“The extrapolated general and specific treatment rates for individuals with alcohol dependence in the total population are shown in Figure 2. Overall, 72.6% [95% CI: 57.8%−94.3%] of the estimated total number of individuals with alcohol dependence and a corresponding ICD-10 diagnosis were registered in the health care system. For 61.9%, no addiction-specific treatments were identified. The share of individuals with at least one of the treatments or care measures considered here was 10.7% [95% CI: 8.4%−13.9%]. Based on the estimate of the overall prevalence and the extrapolation of the routine data, inpatient QWT was initiated by 4.7% [95% CI: 3.7%−6.1%], whereas 4.3% [95% CI: 3.4%−5.5%] used outpatient addiction care services, 3.9% [95% CI: 3.1%−5.0%] used addiction rehabilitation, and 0.8% [95% CI: 0.7%−1.1%] used outpatient drug-based relapse prevention interventions (i.e., anti-craving medications).”

**Figure 1:**
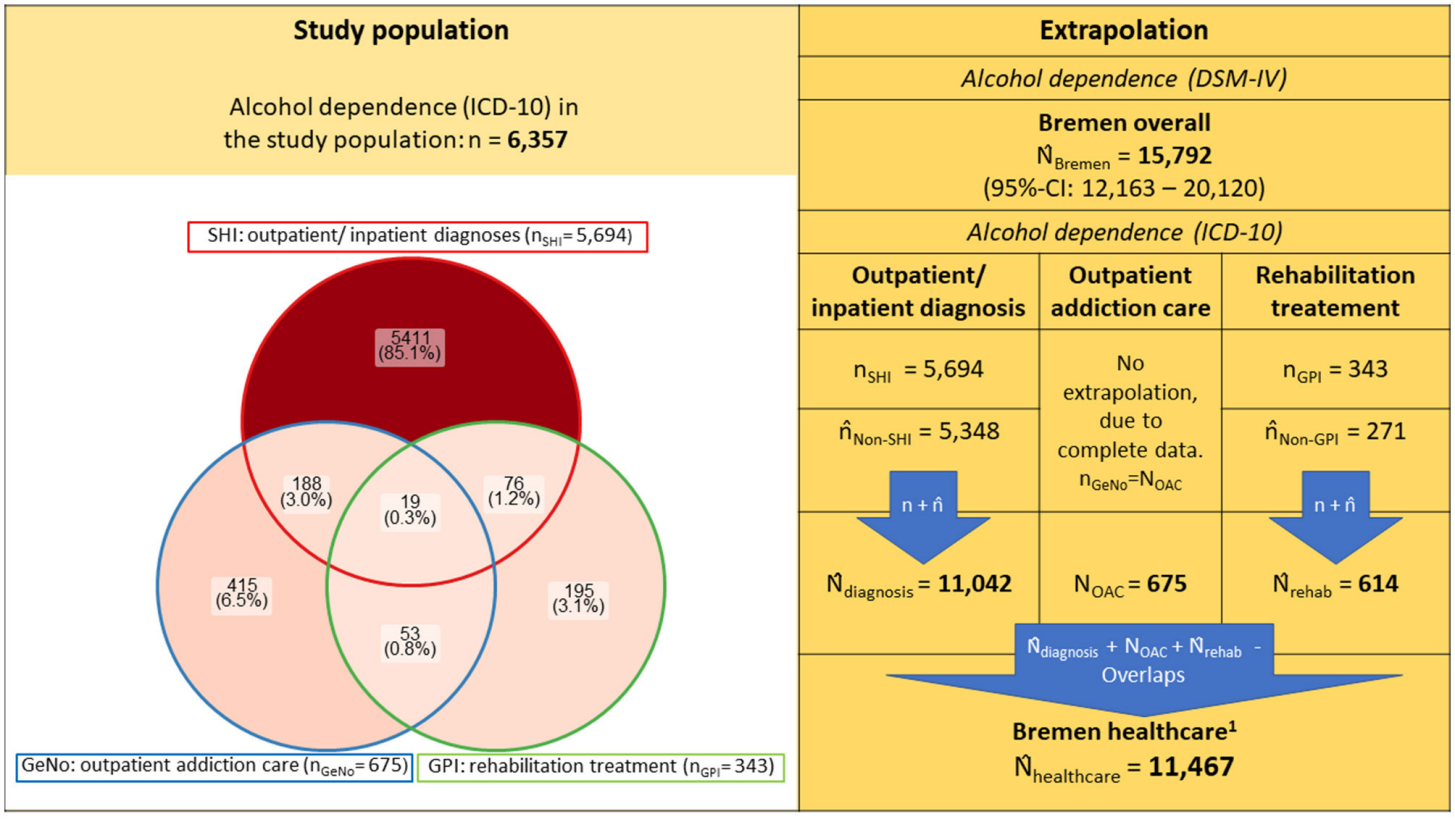
Prevalence of alcohol dependence in study population and extrapolated to total population of Bremen and its caption ^**^n/N denote each the empirical sample and population sizes, whereas n/N represent the estimated and extrapolated population sizes. The study population is represented as a non-proportional Venn diagram using the R package “ggVennDiagram.” For detailed extrapolations, see Supplementary Tables 2–5 and Supplementary Figure 2; SHI, Statutory health insurance; GeNo, Gesundheit Nord - Bremen Hospital Group; GPI, German Pension Insurance 1. Estimated individuals recognized with a diagnosis of alcohol dependence (ICD-10) or addiction specific treatment/care in the healthcare system of Bremen.

**Figure 2:**
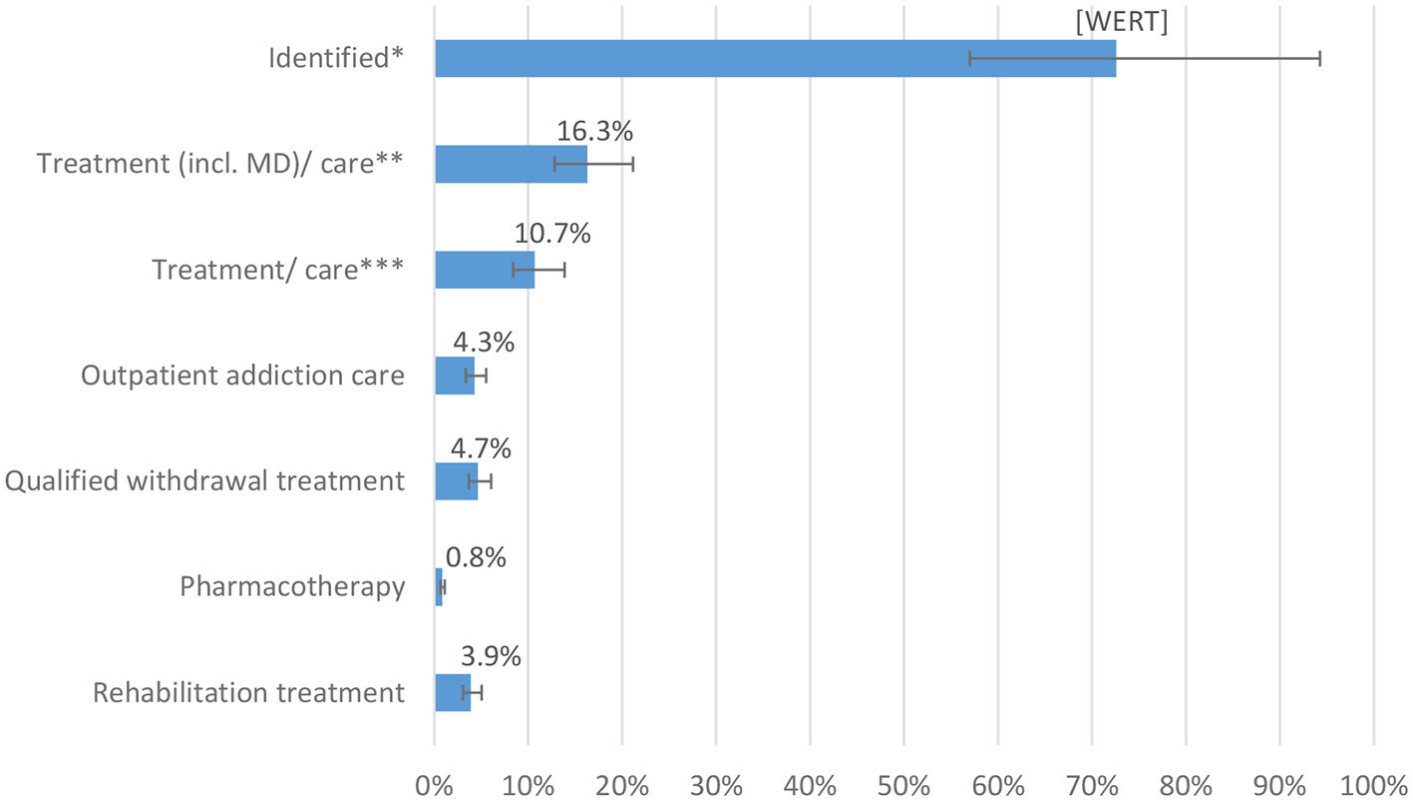
Diagnoses and specific treatment/care rates of persons with alcohol dependence in Bremen 2016/2017 and its caption ^**^Proportions of extrapolated treatments in the estimate for persons with alcohol dependence in the total population of Bremen NBremen 15,792 [12,163–20,120]: ^*^Identified includes individuals with at least one outpatient or inpatient diagnosis, utilization of outpatient addiction care or addiction rehabilitation ^**^Treatment (incl. MD)/care include here: inpatient episode with main diagnosis F10.2–4, qualified withdrawal treatment, pharmacotherapy, outpatient addiction care, and rehabilitation treatment ^***^Treatment/care include here: qualified withdrawal treatment, pharmacotherapy, outpatient addiction care, and rehabilitation treatment.

A correction has been made to 4. Discussion, Paragraph 1. The sentences previously stated:

“The number of individuals with alcohol dependence in the federal state of Bremen in 2017 was estimated at 15,792 (95% CI: 12,163–20,120). Of these, 11,427 persons [72% (95% CI: 57–93%)] received a corresponding ICD diagnosis in medical health care or outpatient addiction care in 2016/2017 and 10% (95% CI: 8–13%) made use of addiction-specific care measures according to our estimates.”

The corrected sentences appear below:

“The number of individuals with alcohol dependence in the Federal State of Bremen in 2017 was estimated at 15,792 [95% CI: 12,163–20,120]. Of these, 11,467 persons (73% [95% CI: 57%−94%]) received a corresponding ICD diagnosis in medical health care or outpatient addiction care in 2016/2017 and 11% [95% CI: 8%−14%] made use of addiction-specific care measures according to our estimates.”

A correction has been made to 4. Discussion, *4.1. Strengths and limitations*, Paragraph 2. This sentence previously stated:

“However, if inpatient main diagnoses of alcohol dependence were considered in calculating the general treatment rate, not only qualified withdrawal but also inpatient physical detoxification would be included (treatment rate with and without inpatient main diagnosis as a treatment: 15.8% vs. 10.0%).”

The corrected sentence appears below:

“However, if inpatient main diagnoses of alcohol dependence were considered in calculating the general treatment rate, not only qualified withdrawal but also inpatient physical detoxification would be included (treatment rate with and without inpatient main diagnosis as a treatment: 16.3% vs. 10.7%).”

A correction has been made to Supplementary Table 5. This table incorrectly included decimal commas in several of the numbers; these have been replaced with decimal points.

The authors apologize for this error and state that this does not change the scientific conclusions of the article in any way. The original article has been updated.

