# Supplementary material for: Estimating the prevalence of alcohol-related disorders and treatment utilization in Bremen 2016/2017 through routine data linkage
Source: Front Psychiatry. 2023 Jan 26;14:1002526. doi: 10.3389/fpsyt.2023.1002526 (PMC9909027; doi:10.3389/fpsyt.2023.1002526)
Supplement: Supplementary file 1 [file Data_Sheet_1.docx]

# Supplementary Material

**e-Figure 1:** Venn diagram of the study population after data linkage.
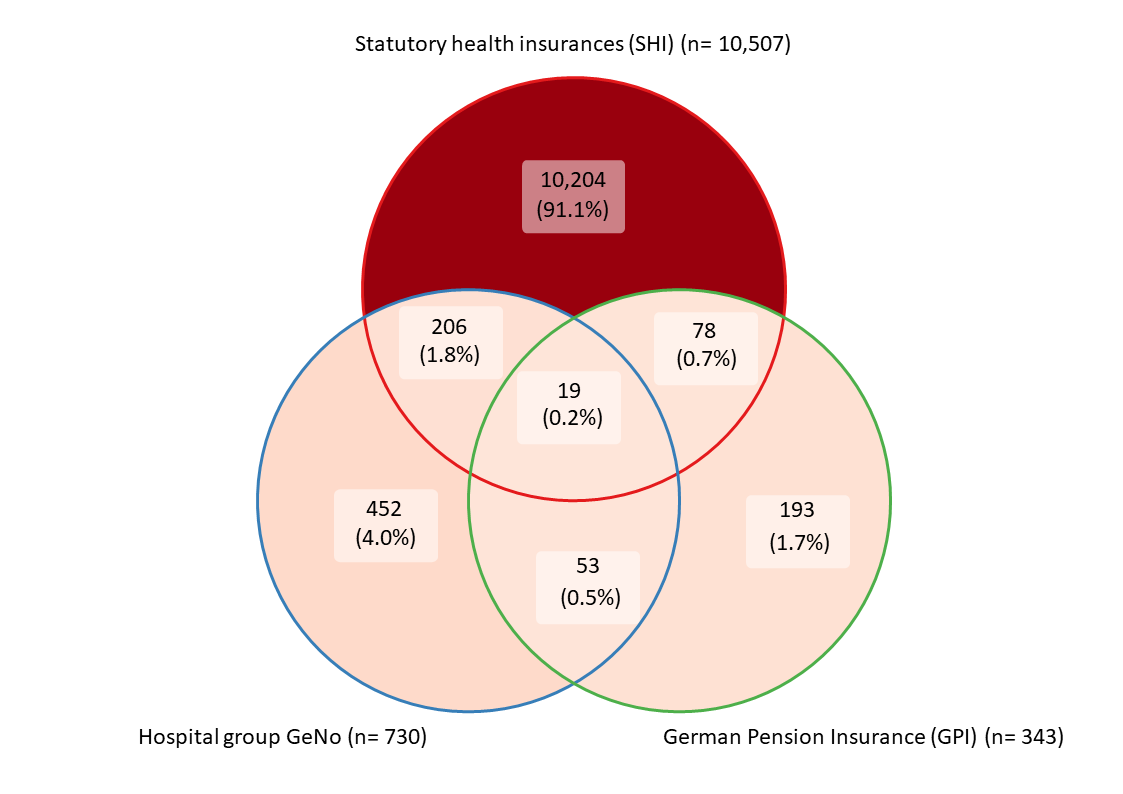


Notes: Individuals who received at least one alcohol-related diagnosis (statutory health insurance (SHI) (AOK and hkk)) and/or attended outpatient addiction care at the Gesundheit Nord - Bremen Hospital Group (GeNo) and/or started an addiction rehabilitation treatment financed by the German Pension Insurance (GPI) in the state of Bremen in 2016/2017. Displayed as a non-proportional Venn diagram using the R package ‘ggVennDiagram’.**e-Figure 2:** Extrapolation to the unobserved populations (not insured in the analyzed statutory health insurances or without health insurance (Non-SHI) and not funded by the German Pension Insurance (Non-GPI)) based on overlaps in the study population with a documented diagnosis of alcohol dependence.
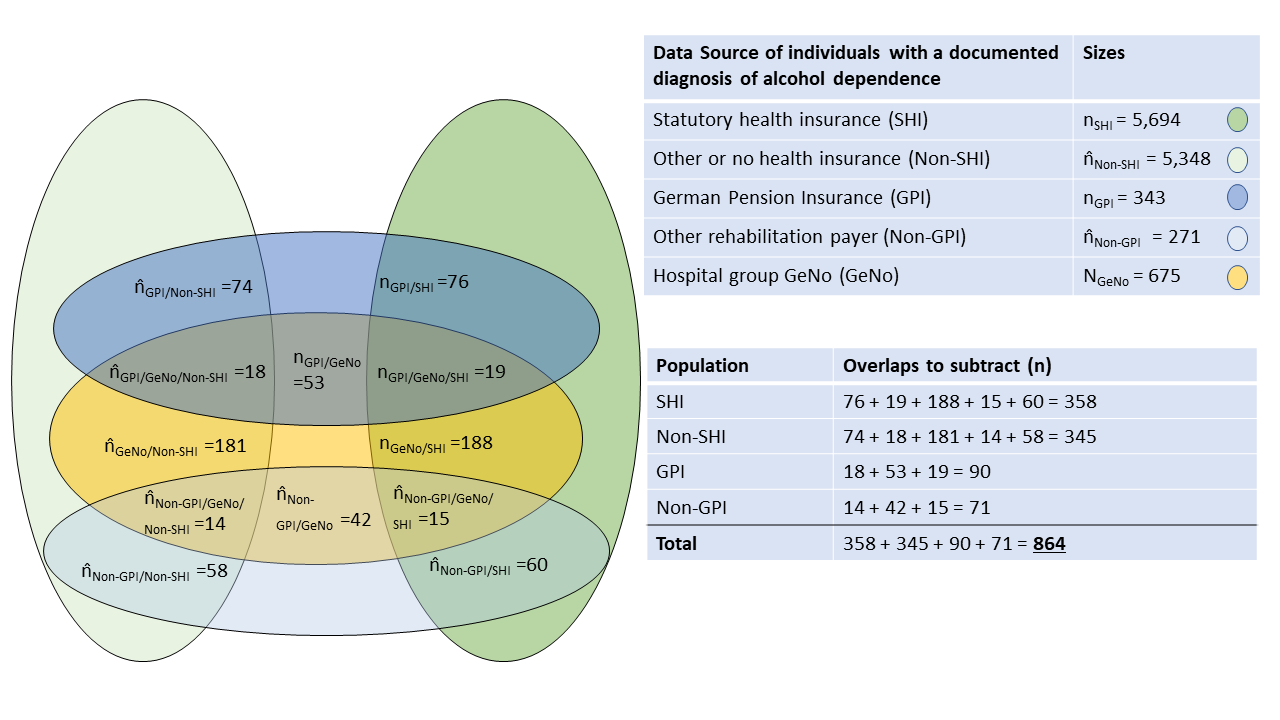


Notes: N/n denotes the empirical sample and population sizes, whereas n̂ represents the estimated and extrapolated population sizes. SHI: Statutory health insurance; GeNo: Gesundheit Nord - Bremen Hospital Group; GPI: German Pension Insurance; For detailed extrapolations see eTable3 and eTable4.

**eTable 1:** Overview of alcohol-related ICD-10 diagnoses

| ICD -10 Codes | Meaning according to ICD-10 |
| --- | --- |
| F10 | Mental and behavioral disorders due to use of alcohol (includes F10.1 to F10.9) |
| F10.0 | Acute intoxication |
| F10.1 | Harmful use |
| F10.2 | Dependence syndrome |
| F10.3 | Withdrawal state |
| F10.4 | Withdrawal state with delirium |
| F10.5 | Psychotic disorder |
| F10.6 | Amnesic syndrome |
| F10.7 | Residual and late-onset psychotic disorder |
| F10.8 | Other mental and behavioural disorders |
| F10.9 | Unspecified mental and behavioural disorder |
| E24.4 | Alcohol-induced pseudo-Cushing syndrome |
| E52 | Niacin deficiency [pellagra] |
| G31.2 | Degeneration of nervous system due to alcohol |
| G62.1 | Alcoholic polyneuropathy |
| G72.1 | Alcoholic myopathy |
| I42.6 | Alcoholic cardiomyopathy |
| K29.2 | Alcoholic gastritis |
| K70.- | Alcoholic liver disease |
| K85.2 | Alcohol-induced acute pancreatitis |
| K86.0 | Alcohol-induced chronic pancreatitis |
| O35.4 | Maternal care for (suspected) damage to fetus from alcohol |
| P04.3 | Fetus and newborn affected by maternal use of alcohol |
| Q86.0 | Fetal alcohol syndrome (dysmorphic) |
| R78.0 | Finding of alcohol in blood |
| T51.- | Toxic effect of alcohol |
| T51.0 | Ethanol |
| T51.9 | Alcohol, unspecified |

**eTable 2:** Extrapolation of individuals with alcohol dependence according to DSM-IV criteria to the total population of Bremen in 2017 using ESA 2018 survey data

|  | **Age** | **N^1^** | **%^2^** | **[95%-CI]** | **N̂_Bremen_^3^** | **N̂_Bremen_ [95%-CI]** |
| --- | --- | --- | --- | --- | --- | --- |
| **Men** | *16-34* | 88,723 | 8.6 | [7.3; 10.1] | 7,630 | [6,476; 8,961] |
|  | *35-64* | 136,420 | 2.5 | [1.7; 3.6] | 3,411 | [2,319; 4,911] |
|  | *65+* | 61,673 | 0.6 | [0.2; 1.1] | 370 | [123; 678] |
| **Women** | *16-34* | 81,293 | 3.6 | [2.9; 4.3] | 2,927 | [2,357; 3,496] |
|  | *35-64* | 134,329 | 0.9 | [0.6; 1.3] | 1,209 | [806; 1,746] |
|  | *65+* | 82,078 | 0.3 | [0.1; 0.4] | 246 | [82; 328] |
| Total | | 584,516 |  |  | 15,792 | [12,163; 20,120] |

Notes: N denote the empirical population size, whereas N̂ represent the estimated population size. The same prevalence of 18–34-year-olds was assumed for 16-17-year-olds. The prevalence of the 65+ age group was estimated from modeling the prevalence of 18-64-year-olds. ^1^ Total population in Bremen 2017; ^2^ Weighted prevalence throughout Germany from ESA 2018; ^3^ Extrapolated total population of individuals with alcohol dependence according to DSM-IV criteria in Bremen; 95% CI = 95% confidence interval.

**eTable 3:** Extrapolation of the administrative prevalence of alcohol dependence (F10.2-4) and addiction-specific care of the statutory health insurances’ (SHI) population 2016/17 to the total population of Bremen

|  |  | | **Bremen^1^** | **SHI^2^** | **Identified^3^** | | | **Outpatient diagnosis** | | | **Qualified withdrawal treatment** | | | **Pharmaco-therapy** | | |
| --- | --- | --- | --- | --- | --- | --- | --- | --- | --- | --- | --- | --- | --- | --- | --- | --- |
|  | **Age** | **N** | | **N** | **n** | **%^4^** | **N̂^5^** | **n** | **%^4^** | **N̂^5^** | **n** | **%^4^** | **N̂^5^** | **n** | **%^4^** | **N̂^5^** |
| **Men** | *16-24* | 37,666 | | 20,061 | 46 | 0.2 | 86 | 30 | 0.1 | 56 | 9 | 0.0 | 17 | 0 | 0.0 | 0 |
|  | *25-49* | 117,355 | | 60,305 | 1,269 | 2.1 | 2,470 | 1,057 | 1.8 | 2,057 | 137 | 0.2 | 267 | 23 | 0.0 | 45 |
|  | *50-64* | 70,122 | | 34,968 | 1,841 | 5.3 | 3,692 | 1,668 | 4.8 | 3,345 | 120 | 0.3 | 241 | 18 | 0.1 | 36 |
|  | *65+* | 61,673 | | 31,691 | 917 | 2.9 | 1,785 | 808 | 2.5 | 1,572 | 19 | 0.1 | 37 | 4 | 0.0 | 8 |
| **Women** | *16-24* | 34,149 | | 17,644 | 25 | 0.1 | 48 | 14 | 0.1 | 27 | 2 | 0.0 | 4 | 0 | 0.0 | 0 |
|  | *25-49* | 110,073 | | 57,042 | 370 | 0.6 | 714 | 327 | 0.6 | 631 | 34 | 0.1 | 66 | 7 | 0.0 | 14 |
|  | *50-64* | 71,400 | | 37,295 | 756 | 2.0 | 1,447 | 699 | 1.9 | 1,338 | 43 | 0.1 | 82 | 11 | 0.0 | 21 |
|  | *65+* | 82,078 | | 48,238 | 470 | 1.0 | 800 | 428 | 0.9 | 728 | 13 | 0.0 | 22 | 5 | 0.0 | 9 |
| Total | | | 584,516 | 307,245 | 5,694 |  | 11,042 | 5,031 |  | 9,754 | 377 |  | 735 | 68 |  | 132 |

Notes: n/N denote each the empirical sample and population sizes, whereas n̂/N̂ represent the estimated and extrapolated population sizes. SHI: Statutory health insurance; GeNo: Gesundheit Nord - Bremen Hospital Group; GPI: German Pension Insurance; ^1^ Total population of Bremen 2017; ^2^ Total population with at least one insurance day in the SHIs AOK or hkk in 2017; ^3^ Diagnosed with an outpatient confirmed or inpatient primary or secondary diagnosis; ^4^ Administrative prevalence of the SHIs’ population; ^5^ Extrapolated to the total population of Bremen.

**eTable 4:** Extrapolation of overlaps from individuals observed in the statutory health insurances’ (SHI) population to people with other or no insurance (Non-SHI) between data on rehabilitation from the German Pension Insurance (GPI) data and data on outpatient addiction care from the hospital group Gesundheit Nord (GeNo)

|  |  | **Bremen^1^** | **SHI^2^** | **Non-**  **SHI^3^** | **Overlaps GPI** | | | **Overlaps GeNo** | | | **Overlaps GPI and GENO** | | | |
| --- | --- | --- | --- | --- | --- | --- | --- | --- | --- | --- | --- | --- | --- | --- |
|  | **Age** | **N** | **N** | **N** | **n_GPI/SHI_** | **%^4^** | **n̂_GPI/Non-SHI_** | **n_GeNo/SHI_** | **%^4^** | **n̂_GeNo/Non-SHI_** | | **n_GPI/GeNo/SHI_** | **%^4^** | **n̂_GPI/GeNo/Non-SHI_** |
| **Men** | *16-24* | 37,666 | 20,061 | 17,605 | 0 | - | 0 | 3 | 0.015 | 3 | | 0 | - | 0 |
|  | *25-49* | 117,355 | 60,305 | 57,050 | 35 | 0.058 | 33 | 78 | 0.129 | 74 | | 11 | 0.018 | 10 |
|  | *50-64* | 70,122 | 34,968 | 35,154 | 36 | 0.103 | 36 | 66 | 0.189 | 66 | | 8 | 0.023 | 8 |
|  | *65+* | 61,673 | 31,691 | 29,982 | 1 | 0.003 | 1 | 18 | 0.051 | 15 | | 0 | - | 0 |
| **Women** | *16-24* | 34,149 | 17,644 | 16,505 | 0 | - | 0 | 0 | - | 0 | | 0 | - | 0 |
|  | *25-49* | 110,073 | 57,042 | 53,031 | 0 | - | 0 | 7 | 0.012 | 7 | | 0 | - | 0 |
|  | *50-64* | 71,400 | 37,295 | 34,105 | 4 | 0.011 | 4 | 13 | 0.035 | 12 | | 0 | - | 0 |
|  | *65+* | 82,078 | 48,238 | 33,840 | 0 | - | 0 | 5 | 0.010 | 4 | | 0 | - | 0 |
| **Total** |  | 584,516 | 307,245 | 277,272 | 76 |  | 74 | 188 |  | 181 | | 19 |  | 18 |

Notes: n/N denote each the empirical sample and population sizes, whereas n̂/N̂ represent the estimated and extrapolated population sizes. SHI: Statutory health insurance; GeNo: Gesundheit Nord - Bremen Hospital Group; GPI: German Pension Insurance;^1^ Total population of Bremen 2017; ^2^ Total population with at least one insurance day in the SHIs AOK or hkk in 2017; ^3^ Total unobserved population in 2017 (N_Bremen_-N_SHI_); ^4^ Proportion of observed individuals on total population N_SHI._

**eTable 5:** Extrapolation of overlaps from German Pension Insurance’s (GPI) data to individuals not observed in the German Pension Insurance’s data (Non-GPI) with observed statutory health insurances’ (SHI) data, unobserved health insurances’ (Non-SHI) data and data from the Gesundheit Nord hospital group (GeNo)

| **Total^1^** | **GPI^2^** | **Non-GPI^3^** | **Overlaps GeNo** | | | **Overlaps Non-SHI** | | | **Overlaps SHI** | | | **Overlaps SHI and GeNo** | | | **Overlaps Non-SHI and GeNo** | | |
| --- | --- | --- | --- | --- | --- | --- | --- | --- | --- | --- | --- | --- | --- | --- | --- | --- | --- |
| **N̂** | **N** | **N̂** | **n_GPI/GeNo_** | **%^4^** | **n̂_Non-GPI/GeNo_** | **n̂_GPI/Non-SHI_** | **%^4^** | **n̂_Non-GPI/Non-SHI_** | **n_GPI/SHI_** | **%^4^** | **n̂_Non-GPI/SHI_** | **n_GPI/GeNo/SHI_** | **%^4^** | **n̂_Non-GPI/GeNo /SHI_** | **n̂_GPI/GeNo/_**  **_Non-SHI_** | **%^4^** | **n̂_Non-GPI/GeNo/_**  **_Non-SHI_** |
| 614 | 343 | 271 | 53 | 15.5 | 42 | 74* | 21.6 | 58 | 76 | 22.16 | 60 | 19 | 5.54 | 15 | 18* | 5.25 | 14 |

Notes: n/N denote each the empirical sample sizes, whereas n̂/N̂ represent the estimated and extrapolated population sizes. ^1^ Extrapolated total population of individuals with initiated rehabilitation treatment in 2016/2017; ^2^ Total population with initiated rehabilitation treatment in 2016/2017 financed by the German Pension Insurance (GPI); ^3^ Total unobserved population with initiated rehabilitation treatment in 216/2017 (N̂_Total_-N_GPI_); ^4^ Proportion of observed/estimated population on total population N_GPI_. *Calculation shown in eTable 4.
